# Diet in Typhoid Fever

**Published:** 1902-07-19

**Authors:** 


### DIET IN TYPHOID FEVER.

At a meeting of the South-Western Branch of the British Medical Association Dr. Norway drew attention afresh to a point which constantly crops up when the subject of typhoid fever is discussed by practical men, namely, the importance of a dietary which shall not leave the intestines charged with undigested and decomposing débris, and the inappropriateness of milk as ordinarily given as a food for such cases. Dr. Norway says that in several necropsies which he made and witnessed on typhoid patients in a London hospital he was struck by the undigested condition of the food found in the stomach and intestines. He has removed from a stomach a mass as large as, and resembling a Spanish onion, which consisted of laminated curdled milk; and in no case did the food found in the stomach or intestines appear to be digested. The food was much as it was before it was swallowed, except that the milk was curdled and the contents of the ileum were very offensive. With these facts before him, together with other theoretical considerations which need not now detain us, Dr. Norway has treated his patients with a mixture by aid of which he hopes to enable them to digest the nourishment given. For an adult the following formula is employed. ℞ Essen. Pepsintiæ (Armour), fl. dr. ½ to fl. dr. i; acid nitro-hydro-chlorici dil., ♏x; glycerini, ♏xx; aquam. ad. fl. oz., ½. This half ounce of solution is to be placed in half a tumbler, or more, of water, and to be sipped or taken as desired to relieve thirst, such a dose being at first given every hour, but as the temperature falls the frequency of the dose being gradually lessened. The mixture, however, is to be given throughout the illness until pain in the iliac fossa disappears and the stools become normal. The diet consists as far as possible of milk (unboiled, but from a carefully selected dairy). To vary the flavour tea or coffee may be added. Five or six ounces of milk may be given every two hours. Occasional meals of some form of beef tea should be given to prevent the patient becoming too tired of milk, and very soon bread and milk, egg and milk, arrowroot, milk puddings, and scrambled eggs may be given, but a daily inspection of the stools can alone decide a change of diet.

